# A Study on Pubmed Search Tag Usage Pattern: Association Rule Mining of a Full-day Pubmed Query Log

**DOI:** 10.1186/1472-6947-13-8

**Published:** 2013-01-09

**Authors:** Abu Saleh Mohammad Mosa, Illhoi Yoo

**Affiliations:** 1 University of Missouri Informatics Institute (MUII), 241 Engineering Building West, Columbia, MO 65211, USA; 2 Health Management and Informatics (HMI) Department, University of Missouri School of Medicine, CS&E Bldg. DC006.00, Columbia 65212 MO, USA

## Abstract

**Background:**

The practice of evidence-based medicine requires efficient biomedical literature search such as PubMed/MEDLINE. Retrieval performance relies highly on the efficient use of search field tags. The purpose of this study was to analyze PubMed log data in order to understand the usage pattern of search tags by the end user in PubMed/MEDLINE search.

**Methods:**

A PubMed query log file was obtained from the National Library of Medicine containing anonymous user identification, timestamp, and query text. Inconsistent records were removed from the dataset and the search tags were extracted from the query texts. A total of 2,917,159 queries were selected for this study issued by a total of 613,061 users. The analysis of frequent co-occurrences and usage patterns of the search tags was conducted using an association mining algorithm.

**Results:**

The percentage of search tag usage was low (11.38% of the total queries) and only 2.95% of queries contained two or more tags. Three out of four users used no search tag and about two-third of them issued less than four queries. Among the queries containing at least one tagged search term, the average number of search tags was almost half of the number of total search terms. Navigational search tags are more frequently used than informational search tags. While no strong association was observed between informational and navigational tags, six (out of 19) informational tags and six (out of 29) navigational tags showed strong associations in PubMed searches.

**Conclusions:**

The low percentage of search tag usage implies that PubMed/MEDLINE users do not utilize the features of PubMed/MEDLINE widely or they are not aware of such features or solely depend on the high recall focused query translation by the PubMed’s Automatic Term Mapping. The users need further education and interactive search application for effective use of the search tags in order to fulfill their biomedical information needs from PubMed/MEDLINE.

## Background

In medical practice, research and education, efficient biomedical bibliographic database (such as PubMed/MEDLINE) search is a core skill for the practice of evidence-based medicine [1-4]. The amount of biomedical information doubles every 5 years [5]. PubMed/MEDLINE, maintained by the National Library of Medicine (NLM), is one of the largest and freely available biomedical bibliographic databases in the world [4-7] and considered as one of the most important and reliable healthcare information source by healthcare professionals [8,9]. PubMed/MEDLINE is also an important source for the literature-based discovery [10]. However, poor query formulation was found to be an obstacle in seeking answers to clinical questions as well as in the practice of evidence-based medicine [11,12].

PubMed/MEDLINE contains citations and abstracts from approximately 5,516 current biomedicine and health related journals, including the fields of medicine, nursing, dentistry, veterinary medicine, health care system and preclinical sciences, from the U.S. and over 80 foreign countries in 39 languages (60 languages for older journals) since 1946 and earlier. There are more than 21 million citations in PubMed/MEDLINE as of November, 2011. About 83% of them are English citations [13,14].

The proper use of search tags (described in the next section) along with search terms is a key for efficient and effective information retrieval in PubMed [15,16]. The main objective of this study was to analyze a typical day’s query log from PubMed in order to discover relationships among PubMed search tags by end users and understand the usage pattern of search tags. For this purpose, the Association Rule Mining (ARM) technique was used.

The analysis of PubMed search tag usage is imperative in terms of information retrieval performance. PubMed users should know and use search tags unlike Google searches. There are two main reasons. First, while PubMed data (i.e., the MEDLINE DB) are well structured (author, paper title, journal, publication date, etc.), web data Google uses are not structured. Thus, one should take advantage of the structure (i.e., using search tags) for PubMed searches for better retrieval performance. Otherwise, a search term is searched in unintended fields causing many irrelevant documents and/or fewer relevant documents (if a search tag is not used in PubMed, a search term is searched in all fields). Second, while Google sorts search results by relevance, PubMed sorts retrieved citations in reverse date added order. In other words, Google’s search results (sorted by relevance) satisfy most users while PubMed’s does not (reverse date added order is not useful to users in most cases).

The NLM recognizes that use of search tags is very important for PubMed searches and, at the same time, PubMed users do not use search tags much. As a result, PubMed has the Automatic Term Mapping (ATM) function that is a search query preprocessing step for novice PubMed users [14]. The ATM analyzes user queries to check if a word or term is structured data such as MeSH terms, author names, journal names, etc. If so, the ATM automatically adds a right search tag to the search term. Search-tag enforced queries by the ATM rather than original user queries are actually for PubMed searches. Because PubMed adopts a recall-focused search mechanism meaning that PubMed attempts to retrieve all relevant documents even though many irrelevant documents are unnecessarily retrieved by the mechanism, the ATM modifies a user query to get each word searched in all fields. Thus, PubMed users should know search tags to understand and/or modify ATM-enhanced queries to meet their information needs (the ATM is a very complex function so refer to [14] for details). Another example showing the NLM wants PubMed users to take advantage of PubMed search tags is its new search result interface. The NLM has recently changed the main PubMed search interface to accommodate (in the left panel of PubMed search result pages) several search tags (e.g., “Publication Type”, “Language”, “Subset”, “Publication Date”) so that PubMed users who are not familiar with or aware of PubMed search tags can instantly apply frequently used search tag(s) to a search result. The new enhanced PubMed interface highlights the need for using search field tags for better PubMed search performance. In summary, using search tags is a crucial factor to improve information retrieval performance in PubMed.

### PubMed/MEDLINE search field tags

PubMed/MEDLINE is a Boolean search system, in which the citations and abstracts are stored in a structured database having many fields or attributes including title, abstract, authors name, journal or proceedings name, publication type, publication date, etc. The citations are indexed in the database with the Medical Subject Headings (MeSH) controlled vocabulary. A set of MeSH terms is applied on every citation that describes the content of the article [14]. Accordingly, searching PubMed/MEDLINE is searching its database fields.

In a PubMed/MEDLINE search query, a search term can be tagged using a database field name enclosed in square brackets that is appended with the search term (e.g., diabetes [Title]). Here, a database field name enclosed in square brackets is called a search field tag that ensures searching of the term in the specified database field only, instead of searching the entire database fields. Tables 1 and 2 present the lists of 48 search field tags in PubMed/MEDLINE.

**Table 1:** **PubMed**/**MEDLINE informational search field tags **[14]

**Search field tag**	**Variants**
[MESH TERMS]	[MH], [MESH]
[MESH MAJOR TOPIC]	[MAJR]
[MESH SUBHEADINGS]	[SH], [SUBHEADING]
[FILTER]^*^	[FILTER]^*^
[LANGUAGE]	[LA], [LANG]
[EC/RN NUMBER]	[RN], [EC], [ECNO]
[OTHER TERM]	[OT], [KEYWORD]
[PS]^*^	[PS]^*^
[SUPPLEMENTARY CONCEPT]	[NM], [SUBS], [SUBSTANCE NAME]
[PHARAMCOLOGICAL ACTION]	[PA]
[PLACE OF PUBLICATION]	[PL]
[PUBLICATION TYPE]	[PT], [PTYP]
[SUBSET]	[SB]
[TEXT WORDS]	[TW], [TEXT], [WORD]
[TITLE]	[TI], [TITL]
[TITLE/ABSTRACT]	[TIAB]
[TRANSLITERATED TITLE]^#^	[TT]^#^
[ALL FIELDS]	[ALL], [ALL FIELD]
COMMENT CORRECTIONS ^#^	N/A

^*^No variation of this tag was observed either in the PubMed documentation [14] or query log file.

^#^This tag did not appear in the user query log file.

**Table 2:** **PubMed**/**MEDLINE navigational search field tags **[14]

**Search field tag**	**Variants**
[AFFILIATION]	[AD], [AFFIL]
[ARTICLE IDENTIFIER]	[AID], [DOI], [PII]
[AUTHOR NAME]	[AUTHOR], [AU], [AU NAME], [AUTH]
[BOOK]^*^	[BOOK]^*^
[CORPORATE AUTHOR]	[CN]
[CREATE DATE]^#^	[CRDT]^#^
[COMPLETION DATE]^#^	[DCOM]^#^
[EDITOR]^#^	[ED]^#^
[ENTREZ DATE]	[EDAT]
[FIRST AUTHOR NAME]	[1AU], [FIRST AUTHOR]
[FULL AUTHOR NAME]	[FAU], [FULL]
[FULL INVESTIGATOR NAME]^#^	[FIR]^#^
[GRANT NUMBER]	[GR]
[INVESTIGATOR]^#^	[IR]^#^
[ISBN]^#*^	[ISBN]^#*^
[ISSUE]	[IP], [ISS]
[JOURNAL]	[TA], [JOUR], [IS], [JO], [JOURNAL NAME]
[LAST AUTHOR]^#^	[LASTAU]^#^
[LOCATION ID]^#^	[LID]^#^
[MESH DATE]	[MHDA]
[MODIFICATION DATE]^#^	[LR]^#^
[NLM UNIQUE ID]	[JID], [NLMID]
OWNER^#^*	N/A
[PAGINATION]	[PG], [PAGE], [PAGE NUMBER]
[PMID]	[UID]
[PUBLISHER]	[PUBN]^#^
[PUBLICATION DATE]	[DP], [PDAT]
[SECONDARY SOURCE ID]	[SI]
[VOLUME]	[VI], [VOLUME NUMBER], [VOL]

^*^ No variation of this tag was observed either in the PubMed documentation [14] or query log file.

^#^This tag did not appear in the user query log file.

A search query that does not contain a search tag or double quotation marks is translated by the Automatic Term Mapping (ATM) in order to improve retrieval performance [17]. In ATM, the untagged terms are matched against the MeSH, journal, author, and investigator translational tables sequentially. If a match is found in one of the translation table, then the term is tagged based on the translation table used. Otherwise, the term is tagged using the “[ALL FIELDS]” tag indicating searching of the term in the entire database fields [14,18]. Although ATM was designed to improve retrieval performance, inappropriate mapping of the search term or search tag may be generated by the ATM leading to a different search result than user’s intent [19-21]. The ATM query translation was implemented such a way to ensure retrieval of all of the relevant articles even though many irrelevant articles are retrieved, which is a higher recall focused strategy at the cost of precision [17,22,23]. As such, query texts consisting of tagged search terms (especially using MeSH) returns better search results (with higher precision) than plain query texts consisting of untagged search terms [24-27].

### PubMed search types

Broder (2002) [28] discussed three kinds of queries in web search: navigational, informational, and transactional. The transactional category does not exist within the context of PubMed/MEDLINE searches, but other two kinds are appropriate [29]. The query that intends to retrieve specific documents is categorized as a navigational query (for example, a query containing author name, journal name and publication year) while the query that intends to fulfill information need is categorized as an informational query (for example, a query containing topical MeSH terms (e.g., *hypertension* [MeSH])) [29]. There are a total of 48 search field tags in PubMed/MEDLINE (Table 1 and 2). The descriptions of the PubMed search tags are available in the PubMed Help web site [14].

A PubMed/MEDLINE search query could be a purely informational query consisting of some informational tags only, a purely navigational query consisting of some navigational tags only, or a mixed query consisting of both of informational and navigational tags. Those mixed queries are intended to retrieve specific documents to satisfy information needs; for example, a query with a MeSH term, journal and year for searching information (specified by the MeSH term) published in a specific journal during a particular year.

### Related works

The study of user searching behavior is very important for user centric design of search engines or digital libraries. There are a number of approaches for studying user searching behavior such as qualitative or quantitative studies, eye-tracking, surveys, server log analysis, etc. The server log analysis has become a viable solution for many applications including search engines [29-35]. A search engine usually stores users’ query texts along with other information in query log files.

Silverstein et al. (1999) [30] studied a large log file from the AltaVista web search engine containing around 285 million user sessions issuing approximately 1 billion query texts. This study summarized that the users mainly type short queries containing three or fewer terms and most of the users only review the first page containing 10 results. They also found that most of the users rarely modify the query texts and submit another query. Jansen et al. (2000) [31] analyzed a query log from the Excite web search engine containing a total of 51,473 queries submitted by a total of 18,113 users and reported that most of the user sessions consist of single query (2 out of 3, i.e. 66%), which is similar to Silverstein’s finding [30].

Biomedical literature search engines such as PubMed have similarity with web search engines in terms of search functionalities, but differ in terms of information sources and contents. The user domain of biomedical information retrieval applications is also different as of the web search engines. For example, the NLM reported in 2002 that most of the PubMed/MEDLINE users (2 out of 3) are health care professionals and scientists whereas the rest of them are the general public [36]. In response, the query log analysis from PubMed/MEDLINE may reveal different user searching behavior than web search engines. Herskovic et al. (2007) [29] took an initiative to analyze a daylong PubMed query log. This study reported some PubMed usage statistics including the number of users, the number of queries per user, the number of sessions per user, commonly used search terms and search field tags, and frequency of term counts. The same daylong dataset has also been used for studying: (1) segmenting PubMed query sessions by identifying related queries [37], (2) the evaluation of PubMed ATM [17], and (3) semantic annotation of PubMed queries [38]. Two studies conducted by Doğan et al. in 2009 [34] and 2010 [35] reported an extensive analysis using a month long and richer query log data from PubMed. Both of the studies [34,35] reported semantic categorization of PubMed queries, proportion of users against number of queries, proportion of queries against number of terms in a query, and many other interesting statistical metrics. This month-long dataset has also been analyzed for: (1) identifying the journals that are related to user search queries [39] and (2) creating a database of queries that is used for automatically producing query suggestions in response to the original user’s input [40]. Both of the datasets used in Herskovic et al. (2007) [29] and Doğan et al. (2009) [34] are publicly available from the NLM. The dataset from Doğan et al. (2009) [34] does not contain the actual user query texts.

In this study we used the same dataset as Herskovic et al. (2007) [29] since it contains query texts as entered by end users (see the section titled “PubMed Query Log Dataset”). This study is different from the 8 studies that used PubMed log data [17,29,34,35,37-40]. The goal of this study is to understand the usage pattern of the PubMed search tags by extracting the tags from the daylong PubMed log file and identifying associations among them (using an association rule mining algorithm). The rationale behind this study is that PubMed retrieval performance highly depends on the usage of search tags. Furthermore, it may reveal an important insight of the search tag usage pattern by end users. This will provide indispensable information for the design requirements of a new literature search system. To the best of our knowledge, this study is the first study on PubMed search field tag usage.

### Association rule mining

Association rule mining (ARM) is a method of identifying associations among a set of items or objects in a database. ARM is also known as frequent itemset mining. The outcome of ARM is association rules, statements of the form *A → B* [*support*, *confidence*]. Here, the support and confidence (user parameter/input) indicate the measures of usefulness and certainty of the rule, respectively. Accordingly, the support and confidence measures are used to filter out uninteresting association rules.

In biomedical research, ARM has unearthed important associations among drugs and diseases [41]. For example, Chen et al. (2003) [42] used ARM to discover co-prescription patterns in the National Health Insurance Research Database (NHIRD) managed by Taiwan National health Insurance. An example association rule the study discovered is {*Muscle relaxants*, *centrally acting drugs*} → {*antacid*} (*support*=*3*.*8*%, *confidence*=*77*.*5*%), which indicates that 77.5% of patients who take muscle relaxants and centrally acting drugs take antacid and the portion of the transactions in the database that meet the association rule is 3.8%. The analysis by Tai & Chiu (2009) [43] on NHIRD database revealed important association of diseases with Attention Deficit/Hyperactivity Disorder (ADHD) that is a highly common chronic behavior condition in childhood. Association mining technique was also found to be useful in text mining [44] and web usage mining [45].

The rationale behind we apply ARM to the PubMed log dataset is our goal to identify hidden associations among PubMed search tags in the dataset and ARM can automatically discover frequently co-occurring search tags. For the ARM mining, a single user query is regarded as a transaction in the database and each search field tag used in a query as an itemset.

## Methods

### PubMed query log dataset

**Figure 1:**
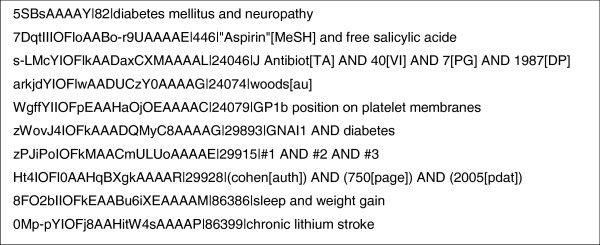
**Sample PubMed query log.** This figure presents a total of 10 sample queries from the PubMed query log file that was used in this study.

The dataset used in the study is a single day’s PubMed query log (which was issued over 24 hours from midnight to midnight) that was obtained from the NLM FTP site (ftp://ftp.ncbi.nlm.nih.gov/pub/wilbur/DAYSLOG, last accessed on 4/21/2012). It is a “|” delimited plain text file and consists of three columns: (1) unique user identification (user ID), (2) timestamp, and (3) query text. The user ID is a set of letters and numbers that was provided in order to match multiple queries from the same user and kept anonymous by removing personally identifying information in order to protect the user privacy. The timestamp presents the time of query submission in number of seconds since midnight EST. The query text is the character string as entered by the user [29]. A total of 2,996,301 queries were recorded in the log file issued by a total of 626,554 distinct users. In Figure 1, a total of 10 sample queries are presented in order to illustrate the content of the query log file. The log file was imported into the Microsoft Access Database for ease of analysis.

### Data pre-processing

**Figure 2:**
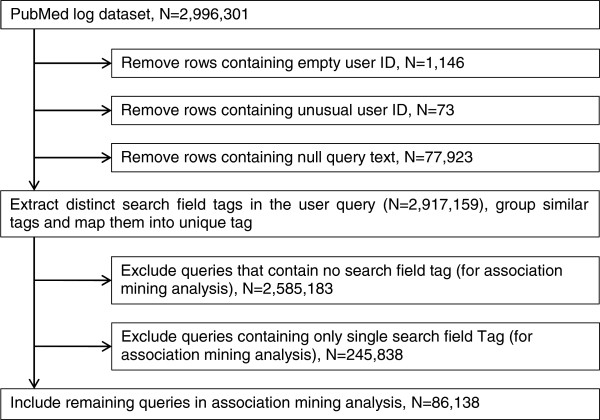
**Data pre-processing steps.** This figure demonstrates the data cleaning and pre-processing steps for association mining analysis.

Figure 2 demonstrates the steps of data cleaning and pre-processing for the association mining analysis. Firstly, the following inconsistencies were identified: (1) 1,146 records (i.e., queries) (0.04%) have no user ID, (2) 73 records (0.0024%) have unusual user IDs that do not comply with the format of majority of user IDs, and (3) 77,923 records (2.6%) have no query text. Those records were removed from the dataset. The remaining records (N=2,917,159, i.e., 97.36%) submitted by a total of 613,061 users (97.85%) were used in this study.

In this study, we primarily analyzed the search tags used in the query text. As such, the query texts were parsed in order to extract the search tags. In order to identify search tags from the queries, we adopted a semi-automatic approach consisting of constructing a search tag list containing search tag headers and their variations in a semi-automatic way, and automatically identifying search tags in queries using the list. The reason behind this (semi-automatic) approach is two-fold. First, for each search tag there are several search tag variations (e.g., for the [Author Name] tag there are [Author], [AU Name], [Auth], and [AU] variations) but these variations are not fully documented (even though they are correctly recognized by the PubMed retrieval system). As a result, we cannot automatically identify search tags from the queries. Thus, we created a PubMed search tag list. Here, search field tags were categorized as either informational (a total of 19 tags presented in Table 1) or navigational (a total of 29 tags presented in Table 2) based on their underlying intent. A list of variants for each search tag is also presented in Tables 1 and 2. This list can be reused for other PubMed log studies. Second, many PubMed queries contain incorrect search tags (including typos, e.g., [JORUNAL]) that are not recognized by the PubMed system but domain experts could correctly recognize and read their intentions. There were 963 unique substrings extracted from the user queries. Among them 129 unique search tags (13.4%) were identified as such tags. We manually corrected them for the search tag analysis.

### Association mining analysis

As the main goal of this study was to analyze the usage pattern of PubMed search field tags in user queries, the dataset was analyzed using association rule mining (ARM) technique. The ARM requires a set of transactions in which each transaction contains a set of items. In this study, a single user query and PubMed search tags were considered as a transaction and items, respectively. The ARM generate association rules of the form *X* → *Y* [*support*, *confidence*], where X and Y are sets of search tags indicating if a user uses the X search tags in a PubMed query, he/she also uses the Y search tags.

The Waikato Environment for Knowledge Analysis (WEKA) software (version 3.6.5) [46] was used for our association mining analysis. WEKA provides several association rule algorithms, such as Apriori [47,48] and FPGrowth [49,50]. Its basic input file format is Attribute-Relation File Format (ARFF) [51]. In order to generate an ARRF file for the association mining analysis, 37 unique search tags found in the PubMed log file were used as the attributes. An attribute value for a record is “Y” if the search tag is present in a query, otherwise the attribute value is “N”. There were 86,138 records in the ARRF file.

## Results

### Search field tag usage

**Figure 3:**
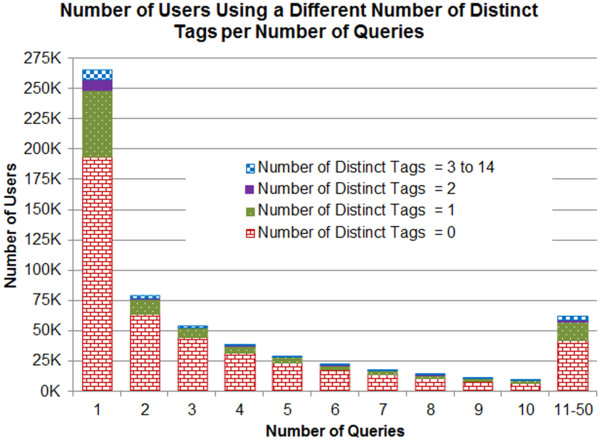
**Number of users using a different number of distinct tags per number of queries.** This histogram presents the total number of users using a different number of distinct search field tags per number of queries.

Table 3 presents the total number of users issuing different number of consecutive queries ranging from 1 to 50. The users issuing more than 50 queries were regarded as institutional proxies or programmatic searches [29]. In response, a total of 2,774 users (0.45%) were excluded from the analysis. The number of users issuing 50 or fewer queries was 610,287 and about two-third (65.51%) of them issued not more than three queries (Table 3). The number of distinct tags used by individual users ranges from 0 to 14. Table 3 also included the number of users issuing a different number of distinct tags. Figure 3 presents the histogram corresponding to Table 3 showing the total number of users using a different number of distinct search field tags (0 to 14) per number of queries.

**Table 3:** Search tag and queries issued per user

**Number of consecutive queries**	**Number of users**				**Total number of users**
	**Number of total distinct tags in the queries**				
	= **0**	= **1**	= **2**	= **3 to 14**	
1	193,935	54,930	9,002	7,758	265,625
2	64,502	12,461	764	1,809	79,536
3	45,023	7,869	561	1,212	54,665
4	31,945	6,016	394	895	39,250
5	24,128	4,634	360	709	29,831
6	18,248	3,898	312	609	23,067
7	14,210	3,267	254	493	18,224
8	11,348	2,703	251	484	14,786
9	9,053	2,295	200	447	11,995
10	7,548	1,966	154	371	10,039
11 to 50	42,741	15,608	1,526	3,394	63,269
Total (%)	462,681	115,647	13,778	18,181	610,287
	(75.81%)	(18.95%)	(2.26%)	(2.987%)	(100%)

This table presents the total number of users using a different number of total distinct tags issuing a different number of queries.

Three out of four users (75.81%) used no search field tag and about two-third of them (65.6%) issued not more than three queries, which represents about half (49.7%) of the total users (Table 3). About one-fifth (19.09%) of the total users issued 4 to 10 queries and 7% issued 11 to 50 queries without using any search field tags. A total of 18.95% of the total users used only one unique search tag; among them 13.31% of the users issued less than four queries and the remaining 5.63% issued more than three queries. 5.24% of the total users used two or more search field tags.

### Search field tag frequency

**Figure 4:**
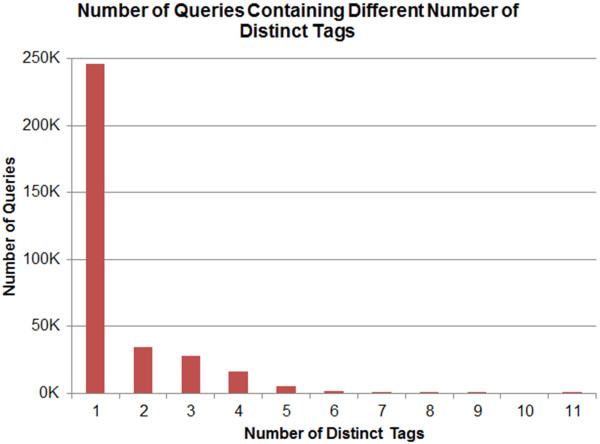
**Number of queries containing different number of distinct tags.** This histogram presents the total number of queries containing different number of distinct tags.

Table 4 presents the total number of queries and its relative frequency for each different number of distinct search field tags, and Figure 4 presents the histogram corresponding to Table 4. The maximum number of distinct tags appeared in a query is eleven. Most of the query texts (N=2,585,183, i.e. 88.62%) did not contain any search field tags (not presented in Figure 4) and 8.43% of the query texts (N=245,838) contained only a single tag.

**Table 4:** Number of queries containing distinct number of tags

**Number of distinct tags**	**Number of queries**	**Relative frequency (%)**
0	2,585,183	88.61989
1	245,838	8.42731
2	34,731	1.19058
3	27,766	0.95182
4	16,320	0.55945
5	5,157	0.17678
6	1,956	0.06705
7	195	0.00668
8	10	0.00034
9	2	0.00007
10	0	0.00000
11	1	0.00003

This table presents the total number of queries (and its relative frequency) containing a different number of distinct tags. The maximum number of distinct tags appear in a query is 11.

**Figure 5:**
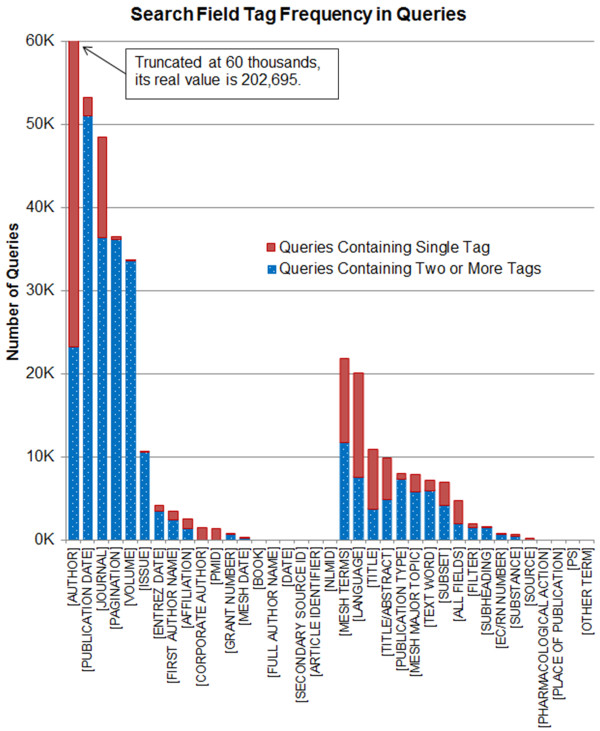
**Search field tag frequency in queries.** This histogram shows for each of 37 search field tags the total number of queries containing either the tag only or the tag and other tag(s).

As explained in the section titled “Data Pre-processing”, the query texts containing two or more search tags were included in the association mining analysis. There were a total of 37 unique search tags appeared in the log file. Among them, 19 tags fall into the navigational category and 18 tags fall into the informational category (Table 5). Table 5 shows, for every search tag, the number of queries containing the tag only and the number of queries with the tag and other tag(s). Figure 5 demonstrates the histogram corresponding to Table 5. This figure (Figure 5) is truncated at 60 thousands in vertical axis for tidy representation. In Figure 5, the navigational tags appeared first in the x-axis followed by the informational tags, and the tags were sorted in decreasing order based on their frequency.

**Table 5:** Search tag frequencies

**Query type**	**Search field tag**	**Number of queries with single tag**	**Number of queries with two or more tags**	**Total**
Navigational	[AUTHOR]	179,418	23,277	202,695
	[PUBLICATION DATE]	2,197	51,021	53,218
	[JOURNAL]	12,153	36,383	48,536
	[PAGINATION]	330	36,213	36,543
	[VOLUME]	89	33,630	33,719
	[ISSUE]	4	10,608	10,612
	[ENTREZ DATE]	695	3,490	4,185
	[FIRST AUTHOR NAME]	1,000	2,478	3,478
	[AFFILIATION]	1,197	1,341	2,538
	[CORPORATE AUTHOR]	1,463	8	1,471
	[PMID]	1,351	65	1,416
	[GRANT NUMBER]	85	652	737
	[MESH DATE]	21	201	222
	[BOOK]	78	1	79
	[FULL AUTHOR NAME]	64	30	79
	[DATE]	13	53	66
	[SECONDARY SOURCE ID]	34	0	34
	[ARTICLE IDENTIFIER]	13	0	13
	[NLMID]	6	0	6
Informational	[MESH TERMS]	10,195	11,704	21,899
	[LANGUAGE]	12,496	7,595	20,091
	[TITLE]	7,180	3,765	10,945
	[TITLE ABSTRACT]	5,001	4,889	9,890
	[PUBLICATION TYPE]	605	7,366	7,971
	[MESH MAJOR TOPIC]	2,047	5,847	7,894
	[TEXT WORD]	1,227	5,950	7,177
	[SUBSET]	2,775	4,167	6,942
	[ALL FIELDS]	2,822	1,922	4,744
	[FILTER]	466	1,564	2,030
	[SUBHEADING]	117	1,552	1,669
	[EC/RN NUMBER]	165	673	838
	[SUBSTANCE]	263	459	722
	[SOURCE]	200	44	244
	[PHARMACOLOGICAL ACTION]	23	50	73
	[PLACE OF PUBLICATION]	23	25	48
	[PS]	19	4	23
	[OTHER TERM]	3	6	9

This table presents the total number of queries containing 37 different search field tags. This table also contains the number of queries containing single tag and two or more tags.

In the navigational category, the most frequently used tag was “[AUTHOR]” followed by “[PUBLICATION DATE]”, “[JOURNAL]”, “[PAGINATION]”, and “[VOLUME]” sequentially (Figure 5). Surprisingly, the [AUTHOR] tag is not very frequently used with other tags (the fifth most frequently jointly used tag). In other words, this tag is usually used alone in a PubMed query. As shown in Figure 5, the informational tags were less frequently used than the navigational tags. The most frequently used informational tag was “[MESH TERMS]” followed by “[LANGUAGE]”, “[TITLE]”, “[TITLE/ABSTRACT]”, “[PUBLICATION TYPE]”, and “[MESH MAJOR TOPIC]”.

One way to significantly improve the performance of PubMed searches is to use MeSH terms along with its search tag [MeSH Terms] or [MeSH] because PubMed documents are indexed with MeSH terms. However, the [MESH TERMS] tag or its variants were explicitly occurred only in 6.6% of the queries (that contained at least one tagged search term) and almost half of them did not co-occur with any other tag. The [MESH TERMS] occurred frequently with [LANGUAGE], [PUBLICATION TYPE], [SUBSET], [MESH MAJOR TOPIC] and [TEXT WORD].

### Search terms vs. search field tags

**Figure 6:**
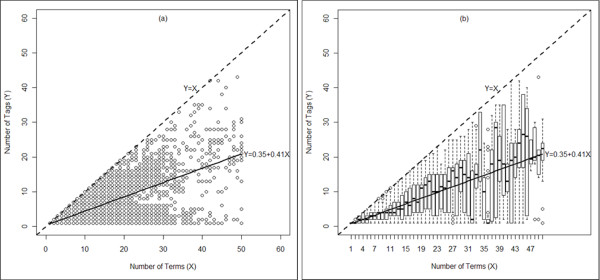
**Plot of tag count against term count: (a) Scatter Plot**, **and (b) Boxplot.** This figure includes a scatter plot diagram and a boxplot diagram presenting the number of search tags (X) against the total number of search terms (Y) used in a query. Also, a linear regression line is superimposed on both of the plot presented by a solid line.

In order to understand the relation between search terms and search tag usage in a query, two diagrams were included: a scatter diagram (Figure 6a) and a boxplot diagram (Figure 6b) presenting the number of search tags (X) against the number of search terms (Y). In Figure 6, a total of 329,061 queries (11.28%) were included satisfying the following criteria: (1) the queries containing one through 50 search terms and at least one tagged search term, (2) the number of search tags is equal or less than the number of search terms thus excluding some erroneous cases, and (3) the query text containing no PubMed history function term. Both of the scatter plot (Figure 6a) and boxplot (Figure 6b) demonstrate that the number of search tags in a query containing at least one tagged search term varied widely regardless of the number of search terms in the query.

We also performed a simple linear regression analysis in order to demonstrate the average usage of search tags in the queries containing at least one tagged search term. Linear regression is a method for modeling the relationship between a dependent variable (*Y*) and one or more independent variable (*X*) in which the conditional mean of *Y* is measured for the given *X*. In this linear regression analysis, we consider the number of search terms in a query as an independent variable (*X*) and the number of search field tags as a dependent variable (*Y*). Since an ideal search query should contain equal number of search terms and search tags, the expected relation between the dependent and independent variable is *Y*=*X*. In both Figure 6a and Figure 6b, the dotted lines through the diagonal (having a slope of 45^*0*^) represent the ideal case.

For the linear regression analysis, we consider the linear equation: *Y* = ∝ *X* + *β* (where ∝ = 1 and β = 0 are expected for the ideal case). We used the R-software for the linear regression analysis [52]. The analysis on the dataset results in an linear equation: *Y* = 0.41*X* + 0.35. The solid line in Figure 6a and Figure 6b represent the linear regression line, which is the conditional mean of *Y* (i.e. the number of search tags) for given *X* (i.e. the number of search terms). The slope of the regression line is *22*.*3*^*0*^, which is almost half of the slope (i.e., *45*^*0*^/*2*) of the ideal case. Thus, we may summarize that the average number of search tags (among the queries containing at least one tagged search term) is almost half of the number of search terms. In other words, on average, half of the search terms are untagged in the queries that contain at least one tagged search term.

### Association mining

The association mining analysis has been done using WEKA to discover frequent co-occurrences of PubMed search field tags. In this association analysis, the minimum support value was 0.02 and the minimum confidence value was 0.50. A total of 104 candidate frequent itemsets were identified satisfying the support requirement. Among them 54 search tags consist of purely informational search tags and the remaining (50) itemsets consist of purely navigational search tags. Interestingly, there were no itemset that consists of both informational and navigational search tags. There were 282 association rules from the frequent itemsets satisfying both of the support and confidence requirements.

**Figure 7:**
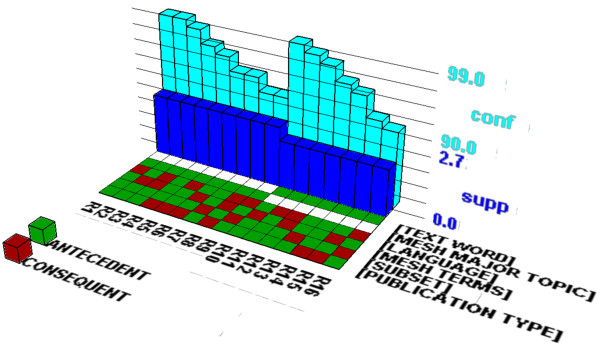
**Visualization of association rules consisting of only informational tags.** This figure visualizes 16 association rules presented in Table 6 consisting of six informational tags (i.e. [LANGUAGE], [MESH TERMS], [PUBLICATION TYPE], [SUBSET], [MESH MAJOR TOPIC], and [TEXT WORD]).

**Figure 8:**
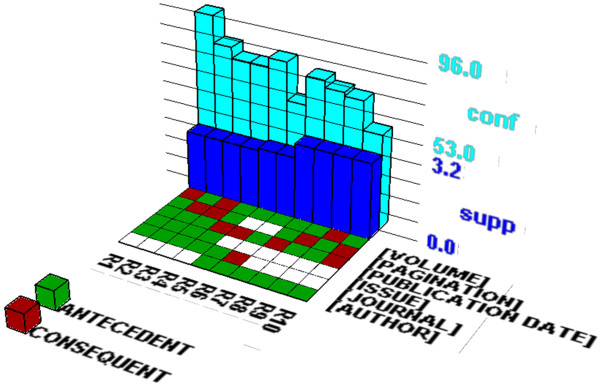
**Visualization of association rules consisting of only navigational tags.** This figure visualizes 10 association rules presented in Table 7 consisting of six navigational tags (i.e. [PUBLICATION DATE], [JOURNAL], [PAGINATION], [ISSUE], [VOLUME], and [AUTHOR]).

We extracted five interesting long itemsets. Table 6 and Table 7 present the association rules consisting of purely informational and navigational tags, respectively. The association rules were visualized (See Figures 7 and 8) using the “Association Rule Viewer (ARV)” software [53]. This novel visualization technique was introduced by Wong et al. (1999) [44]. Originally, it visualized many-to-one association rules (i.e. many items in the antecedent, but only one item in the consequent). However, many association rules are many-to-many so we modified the source code of the software to visualize those rules presented in Tables 6 and 7. In these figures, the associations of search tags are presented in a 2D matrix floor and the support and confidence measures are presented in two bar charts. The rows in the 2D matrix floor present search field tags. Each column in the 2D matrix floor presents an association rule (there are 16 rules). For example, R1 shows the following association rule:

**Table 6:** **Frequent co**-**occurrences of informational search field tags and association rules**

**Itemset No. 1**		**Supp.**
[LANGUAGE], [MESH TERMS], [PUBLICATION TYPE],[SUBSET], [MESH MAJOR TOPIC]		0.027
**Association Rules**	**Conf.**	
[MESH TERMS], [PUBLICATION TYPE], [MESH MAJOR TOPIC], [SUBSET] ==> [LANGUAGE]	0.99	
[LANGUAGE], [PUBLICATION TYPE], [MESH MAJOR TOPIC], [SUBSET] ==> [MESH TERMS]	0.99	
[PUBLICATION TYPE], [MESH MAJOR TOPIC], [SUBSET] ==> [MESH TERMS], [LANGUAGE]	0.98	
[MESH TERMS], [LANGUAGE], [MESH MAJOR TOPIC], [SUBSET] ==> [PUBLICATION TYPE]	0.96	
[MESH TERMS], [MESH MAJOR TOPIC], [SUBSET] ==> [LANGUAGE], [PUBLICATION TYPE]	0.95	
[LANGUAGE], [MESH MAJOR TOPIC], [SUBSET] ==> [MESH TERMS], [PUBLICATION TYPE]	0.93	
[MESH TERMS], [LANGUAGE], [PUBLICATION TYPE], [MESH MAJOR TOPIC] ==> [SUBSET]	0.93	
[MESH MAJOR TOPIC], [SUBSET] ==> [MESH TERMS], [LANGUAGE], [PUBLICATION TYPE]	0.91	
[MESH TERMS], [PUBLICATION TYPE], [MESH MAJOR TOPIC] ==> [LANGUAGE], [SUBSET]	0.91	
**Itemset No. 2**		**Supp.**
[LANGUAGE], [MESH TERMS], [PUBLICATION TYPE],[SUBSET], [TEXT WORD]		0.021
**Association Rules**	**Conf.**	
[LANGUAGE], [PUBLICATION TYPE], [TEXT WORD], [SUBSET] ==> [MESH TERMS]	0.99	
[MESH TERMS], [PUBLICATION TYPE], [TEXT WORD], [SUBSET]==> [LANGUAGE]	0.98	
[PUBLICATION TYPE], [TEXT WORD], [SUBSET] ==> [MESH TERMS], [LANGUAGE]	0.97	
[MESH TERMS], [LANGUAGE], [PUBLICATION TYPE], [TEXT WORD] ==> [SUBSET]	0.95	
[MESH TERMS], [LANGUAGE], [TEXT WORD], [SUBSET] ==> [PUBLICATION TYPE]	0.94	
[LANGUAGE], [TEXT WORD], [SUBSET] ==> [MESH TERMS], [PUBLICATION TYPE]	0.91	
[MESH TERMS], [PUBLICATION TYPE], [TEXT WORD] ==> [LANGUAGE], [SUBSET]	0.9	

This table presents the results of the association mining analysis demonstrating two interesting frequent itemsets consisting of only informational tags. It also presents 16 association rules generated from these two itemsets.

**Table 7:** **Frequent co**-**occurrences of navigational search field tags and association rules**

**Itemset No. 3**		**Supp.**
[PUBLICATION DATE], [JOURNAL], [PAGINATION], [ISSUE], [VOLUME]		0.025
**Association Rules**	**Conf.**	
[PUBLICATION DATE], [JOURNAL], [PAGINATION], [ISSUE] ==> [VOLUME]	0.96	
[PUBLICATION DATE], [JOURNAL], [VOLUME], [ISSUE] ==> [PAGINATION]	0.81	
[PUBLICATION DATE], [JOURNAL], [ISSUE] ==> [PAGINATION], [VOLUME]	0.75	
[JOURNAL], [PAGINATION], [VOLUME], [ISSUE] ==> [PUBLICATION DATE]	0.75	
**Itemset No. 4**		**Supp.**
[JOURNAL], [VOLUME], [AUTHOR], [PUBLICATION DATE]		0.026
Association Rules	**Conf.**	
[JOURNAL], [VOLUME], [AUTHOR] ==> [PUBLICATION DATE]	0.80	
[PUBLICATION DATE], [VOLUME], [AUTHOR] ==> [JOURNAL]	0.59	
**Itemset No. 5**		**Supp.**
[PAGINATION], [VOLUME], [AUTHOR], [PUBLICATION DATE]		0.032
**Association Rules**	**Conf.**	
[PAGINATION], [VOLUME], [AUTHOR] ==> [PUBLICATION DATE]	0.75	
[PUBLICATION DATE], [VOLUME], [AUTHOR] ==> [PAGINATION]	0.71	
[PUBLICATION DATE], [PAGINATION], [AUTHOR] ==> [VOLUME]	0.69	
[VOLUME], [AUTHOR] ==> [PUBLICATION DATE], [PAGINATION]	0.53	

This table presents the results of association mining analysis demonstrating three interesting frequent itemsets comprising of only navigational tags It also presents 10 association rules generated from these three itemsets.

{Publication Type, Subset, MeSH Terms, MeSH Major Topic} → {Language} (Support: 2.7%, Confidence: 99%)

WEKA identified 24 and 22 association rules from the itemsets 1 and 2 respectively. In Table 6, we included the top 9 and 7 association rules from the itemsets 1 and 2 with 90% or higher accuracy (i.e., confidence). The itemsets No. 1 and 2 (consisting of five items) are very similar each other having four common search tags ([LANGUAGE], [MESH TERMS], [PUBLICATION TYPE], [SUBSET]). As a result, they have seven identical association rules. The itemset no. 3, 4, and 5 in Table 7 contain only navigational tags. WEKA discovered 7, 6, and 6 association rules from the itemsets no. 3, 4, and 5, respectively. We included the top 4, 2, and 4 association rules for the itemsets no. 3, 4, and 5, respectively in Table 7. The [AUTHOR] tag, the most frequently used search tag, is included in the itemsets no. 4 and 5 in Table 7.

## Discussion

Search results by experienced PubMed/MEDLINE users using advanced PubMed/MEDLINE features (such as search field tags, Boolean operators, and/or history functions) demonstrated higher recall and precision in earlier studies [54,55]. We believe that the proper use of PubMed search field tags is an important factor in the improvement of PubMed searches. We estimate that only around 11% of PubMed users know how to search PubMed effectively and around 3% of PubMed users are the so-called advanced users because 11.38% of the total queries included a search tag and 25.9% of them (that is 2.95% of the total queries) contained two or more distinct search tags (see Table 5). Around 89% of the PubMed users do not use any PubMed search tag even though using tags in PubMed would significantly improve the quality of information retrieval. We believe there are two reasons. First, many PubMed users are not aware of PubMed search tags. We believe that PubMed should stress the importance of search tags in the website since it does not sort search results by relevance. Although PubMed allows users to use search tags easily through *PubMed Advanced Search Builder*, very few users know the function. Second, many PubMed users believe that PubMed can properly handle their natural language queries like Google so that they think they don’t have to use search tags even if they know them.

PubMed provides a total of 48 search tags (19 informational tags in Table 1 and 29 navigational tags in Table 2). However, only 37 tags were appeared in the query log data including 18 informational and 19 navigational tags presented in Table 5. Not all of these 37 tags were used frequently (Figure 5, Table 5) and only a total of 12 tags (25% of the total search tags) co-occurred frequently with other tags (see Tables 6 and 7). The [AUTHOR] tag was the most frequently used tag in the PubMed queries. Interestingly, it was used mostly alone in PubMed searches. The most frequently used six navigational tags are “[PUBLICATION DATE], [JOURNAL], [PAGINATION], [ISSUE], [VOLUME]”, and [AUTHOR] indicating that many PubMed users search for specific articles using the combinations of these tags. For informational tags we discovered two frequent itemsets as shown in Table 6. Each frequent itemset consists of 5 search tags that are frequently used in PubMed queries. The itemsets share 4 search tags: [LANGUAGE], [MESH TERMS], [PUBLICATION TYPE], and [SUBSET]. Their associations are very strong because the association rules including them have more than 90% confidence. These frequent itemsets can be used for creating an intelligent PubMed search interface. For example, if a user uses one of the four search tags, the PubMed automatically shows or adds the other tags to the query because they are frequently used together so that the user can efficiently compose a PubMed query. Such an intelligent PubMed interface can help users to use PubMed in a more ideal manner.

In the association mining experiment we exploited, the most widely-used association mining algorithm, Apriori in WEKA with the minimum support = 0.02 and the minimum confidence = 0.5. This experiment was conducted on a computer with two Intel Xeon CPUs (at 3.00 GHz) and 24.0 GB RAM. The Apriori algorithm was run for more than five full days consuming more than 20GB system memory, but we were unable to get a result using the algorithm. To tackle this problem, we converted the ARFF input file into a sparse ARFF [51] in which only positive (here, “Y”) values are stored. The sparse format significantly reduced the file size from 6.32 MB to 1.55 MB. Then, we used the FPGrowth algorithm because it was proven to be more efficient than Apriori (while Apriori generates a lot of candidate itemsets, FPGrowth does not) and because, more importantly, it can properly handle a sparse ARFF format meaning that it generates and stores only positive rules containing “Y” values. We got a result (a set of association rules) within 5 seconds (we used the same support and confidence values). We would like to stress that selecting a right data format and algorithm could be critical to successful data mining.

There are two limitations of the study. First, we used only a one-day query log. It is possible that the log could be biased in terms of search tag usage. We had tried to obtain a one-month query log containing user query texts from the NLM that was used in a study by NLM researchers [34] but we could not due to PubMed users’ private issues. Second, we analyzed queries with only search tags. However, most users do not use search tags in their queries, even if they have an intention to search by specific field. Interestingly, many users used untagged search terms along with tagged search term(s), which may result in the user intent of mixed queries containing both of the navigational and informational tags. However, the untagged search terms containing important user intent were not used in the study.

## Conclusions

In this study, a query log of a typical full day from PubMed was studied in order to understand the usage pattern of search tags in PubMed queries. The percentage of search tag usage was low, which suggests that the users do not utilize advanced PubMed search features, they are not aware of such features, and/or they prefer natural language queries to structured queries without considering the structured MEDLINE DB. Further study should be conducted to confirm the reason behind the low usage of search tags. In addition, it has been observed that the frequency of using navigational tags was higher than that of the informational tags. The navigational tags are mainly used in the bibliographic queries.

The results of the association mining demonstrated that the navigational tags and informational tags do not appear frequently together in the same query. The mining result indicates that users are less likely to search both the informational fields and the bibliographic related fields in the same query. Since using search tags is imperative for improving the performance of PubMed searches and most PubMed users do not utilize search tags, there is a great demand for new PubMed search interface that helps users to select appropriate search tag(s) based on our mining results (i.e., sets of frequently associated search tags) for better PubMed searches. The new interface should allow separate customization for each of the informational and navigational categories.

## Competing interests

The authors declare that they have no competing interests.

## Authors’ contributions

ASMM formulated the study design, performed data pre-processing and the experiment, analyzed the experiment results, and drafted the manuscript. IY participated in the study design and interpretation of the experiment results, and finalized the manuscript. All authors read and approved the final manuscript.

## Pre-publication history

The pre-publication history for this paper can be accessed here:

http://www.biomedcentral.com/1472-6947/13/8/prepub
